# Prevalence, Characterization, and Drug Resistance of *Staphylococcus Aureus* in Feces From Pediatric Patients in Guangzhou, China

**DOI:** 10.3389/fmed.2020.00127

**Published:** 2020-04-24

**Authors:** Xiaolan Ai, Fei Gao, Shuwen Yao, Bingshao Liang, Jialiang Mai, Zhile Xiong, Xiantang Chen, Zhuwei Liang, Hongling Yang, Zhiying Ou, Sitang Gong, Yan Long, Zhenwen Zhou

**Affiliations:** ^1^Clinical Laboratory, Guangzhou Women and Children's Medical Center, Guangzhou Medical University, Guangzhou, China; ^2^Clinical Laboratory, Guangzhou Children's Hospital, Guangzhou Medical University, Guangzhou, China; ^3^Clinical Laboratory, Zengcheng Maternity and Children's Health Care Center, Guangzhou Medical University, Guangzhou, China

**Keywords:** *Staphylococcus aureus*, prevalence, characterization, drug resistance, child fecal carriage

## Abstract

**Background:** *Staphylococcus aureus* (*S. aureus*) is a major pathogen of human infections. Its fecal carriage serves as a risk factor for nosocomial transmission and disease development. However, the rate of *S. aureus* fecal carriage among Chinese children has not yet been reported. Therefore, we sought to investigate the prevalence, characterization, and drug resistance of *S. aureus* isolated from pediatric patients' feces in Southern China.

**Methods:** Fecal samples (2059) from pediatric patients in three centers in Guangzhou were cultured. From which, 412 *S. aureus* isolates were identified via selective mediums and automated VITEK Mass Spectrometer analysis. Antibiotic susceptibility was determined and DNA sequencing of seven housekeeping genes were used for multilocus sequence typing analysis.

**Results:** The fecal carriage rates were 20.0% for *S. aureus* and 4.5% for methicillin-resistant *S. aureus* (MRSA). Moreover, *S. aureus* fecal carriage was positively correlated with outpatient status and gastroenteritis diagnosis. Moreover, age-related patterns were observed with respect to prevalence of *S. aureus*. Besides, a total of 76 sequence types (STs) were identified, including 25 newly assigned STs and 28 clonal complexes (CCs). ST188, ST6, and ST15 were the most prevalent methicillin-sensitive *S. aureus* (MSSA) clones, while ST59 and ST45 were the major MRSA clones. *S. aureus* isolates also exhibited high rates of penicillin (84.2%), erythromycin (38.8%), and clindamycin (35.9%) resistance. Specifically, all ST30 and ST338 isolates were resistant to erythromycin and clindamycin, 61% of ST7 were resistant to tetracycline, and 84% of ST45 exhibited resistance and intermediate resistance to rifampicin. Also, CC59 (ST338 and ST59) and CC45 exhibited different antibiotic resistance patterns.

**Conclusion:** These results demonstrate the colonization dynamics and molecular epidemiology of *S. aureus* in child feces in Southern China. Further, they suggest an urgency for strengthening the surveillance programs in China and provide important information for the prevention and treatment of *S. aureus* infection.

## Introduction

*Staphylococcus aureus* (*S. aureus*) is a major pathogen of human infection that causes diseases ranging from minor skin infections to severe bacteremia, necrotizing pneumonia, and life-threatening sepsis (1–3), and thus is a major global threat to human health. *S. aureus* can colonize multifarious body regions, including the anterior nares (4), skin (5), intestinal tract (6), oropharynx (7), and so on. Colonization is a crucial risk factor for the subsequent development of infections (8). Specifically, the importance of *S. aureus* fecal colonization was described as early as 1960 (9), in a study that demonstrated rectal carriage of *S. aureus* earlier than from the nose or throat. Subsequently, several studies have confirmed the clinical importance of *S. aureus* fecal carriage (10, 11). Additionally, *S. aureus* fecal carriage may contribute to environmental contamination (12), which can lead to nosocomial transmission and infection. Previous studies have reported fecal carriage of *S. aureus* in adults from Nigeria (13) and India (14), and a recent study investigated intestinal colonization by *S. aureus* and *Clostridium difficile* in healthy adult fecal samples from China (15), and a few studies have reported on *S. aureus* isolated from pediatric patients' feces samples in China.

Multilocus sequence typing (MLST) has become one of the most popular methods for evaluating *S. aureus* strains; however, only limited MLST studies of *S. aureus* from stool samples are available. Methicillin-sensitive (MSSA) strains ST30, ST398, and ST133 were detected from 100 healthy human fecal samples in Spain (16), while ST15, ST188, and ST59 were identified in six *S. aureus* isolates from stool specimens of diarrheal infants (17). Unfortunately, the diversity of molecular *S. aureus* types in these studies was limited due to the relatively small population size, which may have led to misinterpretation or inaccurate conclusions to be drawn regarding *S. aureus* colonization in fecal samples.

In addition to molecular characterization, antibiotic resistance, notably regarding the emergence and evolution of multi-drug-resistant (MDR) *S. aureus*, has become a major focal point in research across the world. Methicillin-resistant *S. aureus* (MRSA), which begins with resistance to methicillin or most β-lactam antibiotics and gradually develops co-resistance to vancomycin (18, 19), limits the use of alternative anti-infective drugs and threatens patient's health. Hence, drug resistance should be closely monitored to provide the basis for clinical antibacterial infection treatment, including exploring antibiotic resistance of *S. aureus* isolated from fecal samples.

Thus, the aims of this investigation were to evaluate the prevalence, molecular genotyping and antibiotic resistance of *S. aureus* isolated from pediatric patients' fecal samples in Southern China.

## Materials and Methods

### Ethics

All patients were recruited voluntarily and provided informed consent from the participants or the guardians. The study was approved by the research ethics committee of the Guangzhou women and children's medical center (registration no. 2016081029).

### Bacterial Isolates and Data Collection

This study enrolled children from three medical centers in Southern China between August and November 2018, including Guangzhou Women and Children's Medical Center (Tianhe District, central Guangzhou), Guangzhou Children's Hospital (Yuexiu District, western Guangzhou), and Zengcheng Maternity and Children's Health Care Center (Zengcheng District, northern Guangzhou). A total of 2059 non-duplicate pediatric stool samples (1308 outpatients and 751 inpatients) were collected. Approximately 20 mg of stool sample was streaked onto a selective mannitol salt agar medium (Hope Bio-technology, Qingdao, China) within 4 h of sample collection, and incubated in a humidified atmosphere at 37°C with 5% CO_2_ for 24 h. Suspected *S. aureus* colonies from each sample were evaluated based on morphology and sub-cultured on Columbia Blood Agar Medium (Detgerm Microbiology Technology, Guangzhou, China) (20). All isolates were further identified for their species assignment by the automated VITEK MS (bioMérieux, Marcy-l'Étoile, France). Identified *S. aureus* was further confirmed by detecting *femB* (21). We also collected a range of clinical information from the laboratory information system, including gender, age, types, diagnosis, and fecal occult blood test (FOBT). Accordingly, the patients were classified into six age groups: 0–28 days, newborn; 28 days–3 months, young infant; 3 months–1 year, older infant; 1–3 years, child; 3–6 years, pre-school age; 6–18 years, school age and puberty (22, 23). Among these patients, the oldest was 17 years old, the youngest was 1 day, and the median age was 10 months and 23 days.

### Antibiotic Susceptibility Tests

Antibiotic susceptibility for 15 antibiotics (penicillin, oxacillin, gentamicin, rifampicin, levofloxacin, ciprofloxacin, trimethoprim/sulfamethoxazole, clindamycin, erythromycin, macrodantin, linezolid, vancomycin, quinoputin/dafutin, tetracycline, and tigecycline) was detected by VITEK 2 AST-GP67 cards (bioMérieux) using the automated VITEK2 compact system (bioMérieux). Antibiotic minimum inhibitory concentration (MIC) was determined according to the published guidelines (24). The quality control strain used in antibiotic susceptibility analysis was *S. aureus* ATCC 29213. MRSA was defined as an oxacillin-resistant isolate, and multidrug-resistant (MDR) isolates were identified as isolates with resistance to three or more non-β-lactam antibiotics (25).

### DNA Extraction

Total DNA was extracted from 1.0 ml of nutrient broth medium culture grown overnight. After centrifugation, the supernatant was discarded and the *S. aureus* isolates were resuspended in 200 μl of enzymatic lysis buffer (Sangon Biotech, Shanghai, China). Subsequently, *S. aureus* solution was incubated at 37°C for 30 min with 3 μl of lysostaphin (Sigma-Aldrich, Shanghai, China), mixed with 200 μl of Buffer BD (Sangon Biotech), and 200 μl of 100% ethanol (Guangzhou Chemical Reagent Factory, Guangzhou, China), and then transferred to an absorbing column (Sangon Biotech). Next, the Ezup Column Bacterial Genomic DNA Extraction Kit (Sangon Biotech) was used in accordance with the manufacturer's instructions.

### PCR Detection of *femB* and *mecA*

The *femB* gene plays an important role in formatting the pentaglycine bridges that stabilize peptidoglycan chains in *S. aureus*, while the *mecA* gene is the most important cause of *S. aureus* resistance to oxacillin (26). Thus, to further confirm the presence *S. aureus* and MRSA, we detected the expression of *femB* and *mecA* by PCR. The primers used for *femB* and *mecA* genes were described previously (27), and extracted DNA was amplified using TaqTM (Takara, Tokyo, Japan) following the manufacturer's instructions. Following amplification and extension, the PCR amplicons were separated on 1% agarose gels stained with ethidium bromide and visualized under UV illumination (TEX-20 M, Life Technologies, Carlsbad, USA).

### MLST Typing

All isolates were analyzed by multilocus sequence typing (MLST) according to a previously published procedure (28). The PCR products were purified and sequenced by a commercial sequencing company (Beijing Genomics Institute, Shenzhen, China). DNA sequencing of seven housekeeping genes (*arc*C, *aroE, glpF, gmk, pta, tpi*, and *yqiL*) was used for MLST analysis. Sequence types (STs) were determined by searching the *S. aureus* MLST database (https://pubmlst.org/saureus/), which included new emerging MLST alleles and MLST types. Clonal complex (CC) analysis was conducted using the eBURST v.3 programme (https://www.mlst.net/eburst/) according to our previously described protocol (27). Based on STs, a UPGMA dendrogram was constructed with START2.

### Statistical Analysis

Statistical analyses were carried out with SPSS software 20 (SPSS Inc., Chicago, USA). The chi-square (χ^2^) test or Fisher's exact test were applied to dichotomous or categorical variables, which were described as frequencies and proportions. *P* < 0.05 was considered statistically significant.

## Results

### Prevalence of *S. aureus* and MRSA Was Associated With Clinical Features

A total of 2059 fecal samples were collected from pediatric patients in three hospitals, from which 412 *S. aureus* and 93 MRSA isolates were identified. The overall colonization prevalence of *S. aureus* and MRSA were 20.0 and 4.5%, respectively. Accordingly, we classified the 2059 patients into two groups consisting of those that were *S. aureus* positive (*n* = 412) and negative (*n* = 1,647), to analyze the correlation of *S. aureus* fecal carriage with different clinical features. As shown in Table 1, the fecal carriage of *S. aureus* was not associated with patient gender (*P* = 0.149); however, it was positively correlated with outpatient status and gastroenteritis diagnosis (*P* < 0.01). We also observed a positive relationship between *S. aureus* and different age groups. The minimum *S. aureus* carriage rate was in newborn patients (7.3%), while the maximum rate was in infant patients (young infants, 26.6% and older infants 26.3%), after which the carriage rate gradually descended with increasing age of the patients (Table 1). In addition, we observed a positive correlation between FOBT results and *S. aureus* carriage (*P* < 0.01, Table 1).

**Table 1:** Correlation of fecal carriage of *S. aureus* in pediatric patients with different clinical features.

			***S. aureus***			
**Variable**	**Group**	***N***	**+ *N* (%)**	**–*N* (%)**	****χ^**2**^****	***P***
Total patients		2059	412 (20.0)	1647 (80.0)		
Gender	Males	1215	256 (21.1)	959 (78.9)	2.082	0.149
	Females	844	156 (18.5)	688 (81.5)		
Status	Outpatients	1308	344 (26.3)	964 (73.7)	88.643	<0.001^**^
	Inpatients	751	68 (9.1)	683 (90.9)		
Diagnosis	Gastroenteritis	1242	319 (25.7)	923 (74.3)	62.974	<0.001^**^
	Others	817	93 (11.4)	724 (88.6)		
Age	0–28 days	205	15 (7.3)	190 (92.7)	55.629	<0.001^**^
	28 days−3 months	241	64 (26.6)	177 (73.4)		
	3 months−1 year	726	191 (26.3)	535 (73.7)		
	1–3 years	484	85 (17.6)	399 (82.4)		
	3–6 years	240	35 (14.6)	205 (85.7)		
	6–18 years	163	22 (13.5)	141 (86.5)		
FOBT	Positive	784	210 (26.8)	574 (73.2)	36.319	<0.001^**^
	Negative	1275	202 (15.8)	1073 (84.2)		

*+: S. aureus-positive group; –: S. aureus-negative group. Clinicopathological features were assessed using the chi-square test*,

** *P < 0.01*.

Subsequently we divided the 412 positive *S. aureus* patients into two groups, MRSA (*n* = 93) and MSSA (*n* = 319), to explore the relationship of MRSA and clinical features. However, we found that MRSA was significantly correlated with inpatient status (*P* = 0.002), and not gender, age, or FOBT results (Table 2).

**Table 2:** Correlation of MRSA in pediatric patients' fecal samples with different clinical features.

**Variable**	**Groups**	***S. aureus***	**MRSA**	**MSSA**	**χ^2^**	***P***
Total patients		412	93 (22.6)	319 (77.4)		
Gender	Males	256	60 (23.4)	196 (76.6)	0.289	0.591
	Females	156	33 (21.2)	123 (78.8)		
Status	Outpatients	344	68 (19.8)	276 (80.2)	9.385	0.002^**^
	Inpatients	68	25 (36.8)	43 (63.2)		
Age	0–28 days	15	5 (33.3)	10 (66.7)	1.763	0.881
	28 days−3 months	64	15 (23.4)	49 (76.6)		
	3 months−1 year	191	39 (20.4)	152 (79.6)		
	1–3 years	85	20 (23.5)	65 (76.5)		
	3–6 years	35	8 (22.9)	27 (77.1)		
	6–18 years	22	6 (27.3)	16 (72.7)		
FOBT	Positive	210	40 (19.0)	170 (81.0)	3.045	0.081
	Negative	202	53 (26.2)	149 (73.8)		

*MRSA, methicillin-resistant S. aureus; MSSA, methicillin-susceptible S. aureus. Clinicopathological features were assessed using the chi-square test*,

** *P < 0.01*.

### Antibiotic Susceptibility of *S. aureus* and MRSA

The antibiotic susceptibility results for the 412 *S. aureus* isolates according to MLST are presented in Table 3. All 93 MRSA strains were resistant to cefoxitin screening and carried the *mec*A gene. The *S. aureus* strains exhibited highest rate of resistance to penicillin (PEN, 84.2%), followed by erythromycin (ERY, 38.8%), clindamycin (CLI, 35.9%), tetracycline (TE, 14.6%), and sulfamethoxazole-trimethoprim (SXT, 6.1%). The resistance rates of antibiotics were lower for gentamicin (GEN, 2.7%), levofloxacin (LEV, 1.9%), ciprofloxacin (CIP, 1.9%), and rifampicin (RIF, 0.7%); however, all isolates were susceptible to macrodantin, linezolid, vancomycin, dalfopristin/quinupristin (QDA), and tigecycline. Compared to the MSSA group, the MRSA group had significantly higher rates of resistance to PEN (*P* < 0.01), ERY (*P* < 0.01), CLI (*P* < 0.01), and TE (*P* = 0.03) and intermediate resistance to RIF (*P* < 0.01). Although 22.8% of all strains exhibited MDR, the MRSA group had a significantly higher rate (74.2%) compared to that of the MSSA group (7.8%) (*P* < 0.01).

**Table 3:** ntibiotic susceptibility of S. aureus and MRSA isolates from pediatric patients' feces.

**Antibiotic**	***S. aureus*** **(*****n*** **=** **412)**		**MRSA (*****n*** **=** **93)**		**MSSA (*****n*** **=** **319)**		***P*^***a***^**
	**R, *n* (%)**	**I, *n* (%)**	**R, *n* (%)**	**I, *n* (%)**	**R, *n* (%)**	**I, *n* (%)**	
Penicillin	347 (84.2)	0 (0.0)	93 (100)	0 (0.0)	254 (79.6)	0 (0.0)	<0.01^**^
Gentamicin	11 (2.7)	4 (1.0)	2 (2.2)	2 (2.2)	9 (2.8)	2 (0.6)	1.00
Rifampicin	3 (0.7)	43 (10.4)	2 (2.2)	27 (29.0)	1 (0.3)	17 (5.3)	<0.01^b^^**^
Levofloxacin	8 (1.9)	0 (0.0)	4 (4.3)	0 (0.0)	4 (1.3)	0 (0.0)	0.148
Ciprofloxacin	8 (1.9)	1 (0.2)	4 (4.3)	0 (0.0)	4 (1.3)	1 (0.3)	0.149
SXT	25 (6.1)	0 (0.0)	2 (2.2)	0 (0.0)	23 (7.2)	0 (0.0)	0.072
Clindamycin	148 (35.9)	0 (0.0)	67 (72)	0 (0.0)	81 (25.4)	0 (0.0)	<0.01^**^
Erythromycin	160 (38.8)	1 (0.2)	67 (72)	0 (0.0)	93 (29.2)	1 (0.3)	<0.01^**^
Macrodantin	0 (0.0)	0 (0.0)	0 (0.0)	0 (0.0)	0 (0.0)	0 (0.0)	NA
Linezolid	0 (0.0)	0 (0.0)	0 (0.0)	0 (0.0)	0 (0.0)	0 (0.0)	NA
Vancomycin	0 (0.0)	0 (0.0)	0 (0.0)	0 (0.0)	0 (0.0)	0 (0.0)	NA
QDA	0 (0.0)	0 (0.0)	0 (0.0)	0 (0.0)	0 (0.0)	0 (0.0)	NA
Tetracycline	60 (14.6)	0 (0.0)	20 (21.5)	0 (0.0)	40 (12.5)	0 (0.0)	0.03^*^
Tigecycline	0 (0.0)	0 (0.0)	0 (0.0)	0 (0.0)	0 (0.0)	0 (0.0)	NA

*SXT, trimethoprim/sulfamethoxazole; QDA, dalfopristin/quinupristin. R, Resistant; I, Intermediate; NA, not applicable*.

a Antibiotic resistance of MRSA vs. MSSA by chi-squared test (two-sided);

b *Rifampicin intermediate of MRSA vs. MSSA by chi-squared test (two-sided)*,

** *P < 0.01*,

* *P < 0.05*.

### Molecular Characterization of *S. aureus*

**Figure 1:**
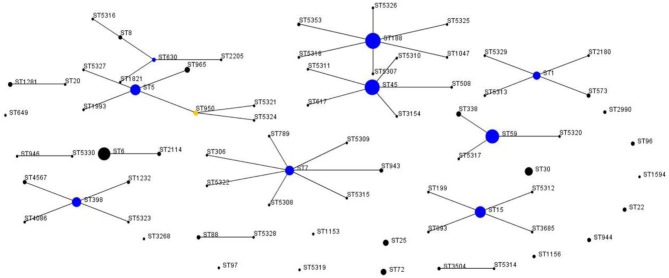
Auto-edited eBURST diagram of 412 *S. aureus* isolates based on the MLST data. The auto-edited eBURST diagram produced using 6/7 group definition shows 76 STs, including 14 groups and 14 singletons. Each dot implies an MLST ST and the dot area indicates the prevalence of the ST in the MLST data of this study. The linked clusters within the population snapshot should represent clonal complexes, and the primary founders and subgroup founders of these linked clusters are colored blue and yellow.

**Figure 2:**
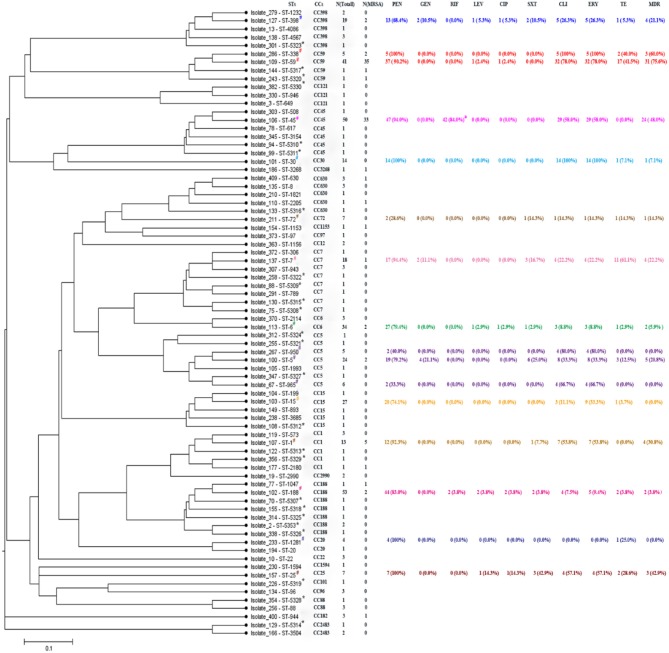
START2 analysis, genotypes, and drug resistances of *Staphylococcus aureus* isolates. STs, sequence types; CCs, clonal complexes; Each ST randomly selected one isolate as shown above. PEN, penicillin; GEN, gentamicin; RIF, rifampicin; LEV, levofloxacin; CIP, ciprofloxacin; SXT, sulfamethoxazole-trimethoprim; CLI, clindamycin; ERY, erythrocin; TE, tetracycline; MDR, multidrug resistance, representing antibiotic resistance of non β-lactamase. *Representing newly assigned STs. ^**#**^Representing 16 STs that each had more than three strains. ^a^Resistance and intermediary resistance of RIF. Different colors represent different clonal complexes.

According to the results of the MLST method, 76 unique STs were identified among 412 *S. aureus* isolates, including 25 novel STs. Based on eBURST analysis, the 76 STs were classified into 28 CCs, including 14 groups and 14 singletons (Figures 1, 2). The three most abundant STs among all *S. aureus* isolates were ST188 (12.9%), ST45 (12.1%), and ST59 (10.0%), comprising 35% of all isolates. Among the MRSA group, the three most abundant STs were ST59 (37.6%), ST45 (35.5%), and ST1 (5.4%), comprising 78.5% of all strains. Among MSSA group, ST188 (16.0%), ST6 (9.7%), and ST15 (8.5%) were the three prevalent STs. Further, the most common CCs among all strains were CC188, CC45, and CC59, representing 39.6% of all clones. Specifically, within the MRSA group, CC59 (41.9%), CC45 (35.5%), and CC1 (6.5%) were the three most abundant CCs, while in the MSSA group, the most common clone was CC188 (17.9%), followed by CC5 (11.6%) and CC6 (11.0%) (Table 4).

**Table 4:** Genotype ranking of *S. aureus* isolated from pediatric patients.

**Rank**	***S. aureus*** **(*****n*** **=** **412)**				**MRSA (*****n*** **=** **93)**				**MSSA (*****n*** **=** **319)**			
	**MLST**	***N* (%)**	**CCs**	***N* (%)**	**MLST**	***N* (%)**	**CCs**	***N* (%)**	**MLST**	***N* (%)**	**CCs**	***N* (%)**
1	ST188	53 (12.9)	CC188	60 (14.6)	ST59	35 (37.6)	CC59	39 (41.9)	ST188	51 (16.0)	CC188	57 (17.9)
2	ST45	50 (12.1)	CC45	55 (13.3)	ST45	33 (35.5)	CC45	33 (35.5)	ST6	31 (9.7)	CC5	37 (11.6)
3	ST59	41 (10.0)	CC59	48 (11.7)	ST1	5 (5.4)	CC1	6 (6.5)	ST15	27 (8.5)	CC6	35 (11.0)
4	ST6	34 (8.3)	CC5	39 (9.5)	ST188	2 (2.2)	CC188	3 (3.2)	ST5	22 (6.9)	CC15	31 (9.7)
5	ST15	27 (6.6)	CC6	37 (9.0)	ST6	2 (2.2)	CC5	2 (2.2)	ST45	17 (5.3)	CC7	26 (8.2)
6	ST5	24 (5.8)	CC15	31 (7.5)	ST5	2 (2.2)	CC6	2 (2.2)	ST398	17 (5.3)	CC398	24 (7.5)
7	ST398	19 (4.6)	CC7	27 (6.6)	ST398	2 (2.2)	CC398	2 (2.2)	ST7	17 (5.3)	CC45	22 (6.9)
8	ST7	18 (4.4)	CC398	26 (6.3)	ST338	2 (2.2)	CC630	2 (2.2)	ST30	14 (4.4)	CC30	14 (4.4)
Total		266 (64.6)		323 (78.4)		83 (89.2)		89 (95.7)		196 (61.4)		246 (77.1)

As shown in Figure 2, there were 25 newly assigned STs (ST5307 to ST5330, and ST5353) in this study, of which many were single-locus variants (SLVs). Among the 25 novel STs, 26 strains were identified, including 2 MRSA and 24 MSSA isolates; both MRSA isolates belonged to CC59, and the most abundant CC in the MSSA groups was CC188. Finally, we identified 18 novel SLVs in seven housekeeping genes, which were subsequently assigned as new alleles (Table S1).

### Association of Antibiotic Resistance With Specific *S. aureus* Sequence Types

When analyzing the correlation between antibiotic resistance profiles and unique sequence types (STs) in genotypes of the *S. aureus* isolates, 16 STs each had more than three strains that were selected in this study. Some specific STs were determined to be closely associated with certain antibiotic resistance patterns, while some exhibited high sensitivity. As shown in Figure 2 and Figure S1, all ST338, ST25, ST30, and ST1281 isolates were resistant to PEN, while ST72, ST950, and ST965 showed high sensitivity to PEN. However, ST950 and ST965 showed high resistance to CLI and ERY, yet were sensitive to all other antibiotics, with an MDR rate of 0%, similar to the MDR rate of ST1281 and ST15. Similarly, ST188, with the largest number of strain types and largest antibiotic resistance coverage, had a very low MDR rate (3.8%). All ST30 and ST338 isolates were resistant to ERY and CLI. ST59, which belongs to CC59 with ST338, also showed a high resistance rate (78%) to ERY and CLI. Additionally, ST59 and ST338 had a higher rate of resistance to TE, with the two highest MDR rates of 75.6 and 60.0%, respectively. In addition to the STs mentioned above, ST7 exhibited the highest resistance rate (61.1%) to TE, while ST45, another predominant ST in the MRSA isolate group, had the highest resistance and intermediate resistance rate (84.0%) to RIF (Figure 2 and Figure S1).

## Discussion

*S. aureus* fecal carriage may contribute to environmental contamination, facilitate nosocomial transmission, and promote human disease development. Moreover, fecal carriage of *S. aureus* among children is more likely to cause disease infection due to their immature and underdeveloped immune system (29). Since *S. aureus* fecal colonization has been identified as a risk factor for infection disease development (30), our findings may serve to advance the current understanding regarding *S. aureus* fecal colonization dynamics and prevention of *S. aureus* infection.

In this study, the *S. aureus* fecal carriage rate was determined to be 20.0% in pediatric patients from Guangzhou, while the MRSA carriage rate was 4.5%. These results agree with the reported prevalence of pooled estimates for *S. aureus* and MRSA fecal carriage rates (16.8–36.3%, 0.7–27.0%, respectively) (30). However, the prevalence of *S. aureus* in this study was higher than that reported from participants with nosocomial diarrhea in Germany (7%) (31) compared to healthy adults in China (3.51%) (15), but was lower than that reported in a previous Nigerian study (31.7%) (13). Moreover, in China, the prevalence of MRSA nasal colonization in children between 2005 and 2015 was 4.4% (32), similar to the 4.5% carriage rate detected in our study, but slightly higher than a previous study in American children with cancer (2.9%) (33). Although the prevalence of *S. aureus* and MRSA is dynamic due to differences in geographical regions, age, gender, and health status, future studies continue to be warranted to better characterize the cause of these differences.

In our study, the fecal carriage of *S. aureus* was positively correlated with outpatient status and gastroenteritis diagnosis. Pediatric gastroenteritis primarily manifests as abdominal pain, diarrhea, and vomiting, and these patients comprise the majority of outpatients. Moreover, *S. aureus* has been described as the most common global causative pathogen of food-borne illness, while studies have reported it to be associated with infantile diarrhea (17), corresponding with our observed positive correlation between *S. aureus* fecal carriage and gastroenteritis in children. In addition, our study found that fecal *S. aureus* colonization was lowest during the first 4 weeks of life, after which it increased rapidly during the first year, followed by a gradual decline until 17 years of age. This may be explained by the underdeveloped intestinal function and microbiota composition in newborn patients. With improved intestinal function and increased diversity of intestinal microbes, *S. aureus* colonization increased within the following year. Further, human milk oligosaccharides have been suggested to be a strong contributor to bacterial reproduction in the infant gut and to stimulate *S. aureus* growth (34), providing an important function for breastfeeding in early life. Subsequently, with the consumption of a comprehensive diet and enhanced immune function, the fecal carriage of *S. aureus* decreases with increasing age. Additionally, within this study, *S. aureus* fecal carriage was higher in patients that tested FOBT positive, which may be explained by virulence factors, especially staphylococcal enterotoxins, produced by *S. aureus*, causing intestinal damage that leads to intestinal bleeding. However, this hypothesis requires further validation.

We also determined that fecal carriage of MRSA was positively associated with inpatients status as hospitalized patients are more likely to be infected with MRSA (35). Interestingly, 7 of the 68 hospitalized patients with *S. aureus* in their stool also had it within in their sputum or alveolar lavage fluid, demonstrating similar antibiotic susceptibility patterns, including 2 MRSA and 5 MSSA isolates (data not shown). This may suggest that the *S. aureus* isolated from stool sample was consistent with the source in the sputum or alveolar lavage fluid, and fecal carriage of *S. aureus* may be associated with infection in other parts of the body.

Based on the MLST results, 412 *S. aureus* isolates were divided into 76 STs, with fewer MRSA isolates (14 STs) than MSSA isolates (69 STs), indicating that MRSA isolates were more genetically stable than MSSA isolates. The most commonly reported *S. aureus* isolates in China are diverse and include ST1 (36), ST6 (20), ST5 (37), and ST188 (38) according to different regions, ages, and resources. Similarly, many different MSSA isolates have been identified throughout China, including ST7 and ST188 (39). In our study, ST188, ST6, and ST15 were the most frequently observed STs in the MSSA group. ST188 was reported as a major cause of childhood infections in China, due to its high adhesion and biofilm formation ability (38). In addition, ST15 and ST188 were the two most prevalent clones isolated from infantile diarrhea fecal samples (17). Although the types of MSSA strains are diverse, the most abundant ones have not changed significantly among children in China.

MRSA strains demonstrated strong homology with the prevalent clones in this study, which were determined to be ST59 (37.6%) and ST45 (35.5%). This result was consistent with a previous study reported by Ding et al. in Chinese children (40). ST59, a predominant MRSA clone causing CA-MRSA infections among children (41), was not only predominant in Chinese cities, including Shanghai (39), Sichuan (42), and Taiwan (43), but also is considered to be a prevalent isolate throughout the Asia-Pacific region (44). It is worth noting that ST45, the second most prevalent MRSA strain, has an increased carriage rate in children, compared to 18.8% for the MRSA isolated in our previous study (27). ST45, known as a Berlin clone, was also a common isolate throughout European countries, and has now spread to Australia (45), Singapore (46), and China (41). Further, previous studies from Shanghai have shown that ST239 was the most frequent MRSA clone between 2005 and 2010 (47), which was replaced by the increasingly abundant ST5, ST59, and ST398 clones between 2008 and 2017 (48). However, ST239 was not identified in our study, and ST5 and ST398 only accounted for 2.2% of MRSA isolates. Alternatively, ST45 and ST59 were not only the two dominant clones in the MRSA group, but also the second and the third most abundant clones in all *S. aureus* isolates. ST45-MRSA is attributed to the acquisition of *mecA* by a MSSA clone in the community (49). Therefore, our results suggest that ST45 would alter the MRSA clone structure in this region of China, allowing for the development of additionally prominent clones in Chinese children, such as ST59.

The drug resistance of *S. aureus* has attracted great attention worldwide, especially in China, due to the abuse of antibiotics and the recent emergence of MDR bacteria. Consistent with a previous report (50), we show that *S. aureus* isolates exhibited a high rate of resistance to PEN, ERY, and CLI, which may be the result of the excessive use of PEN and macrolides (51). The current study demonstrated that *S. aureus* was more susceptible to vancomycin and linezolid, and displayed 100% sensitivity to vancomycin, linezolid, macrodantin, and QDA. Similarly, relatively low rates of resistance to CIP and LEV were observed in *S. aureus* isolates, which may be a result from the infrequent use of fluoroquinolones in pediatric patients due to their reported cartilage toxicity. In addition, *S. aureus* strains can differ in their antibiotic resistance patterns with specific STs. CC59 (ST59 and ST338) exhibited the highest MDR rate and a specific antibiotic resistance pattern (ERY-CLI-TE), while another common clone, CC45 (ST45), exhibited a different antibiotic resistance pattern (ERY-CLI-RIF). ST45 resistance or intermediate resistance to RIF is caused by *rpo*B mutations (52). However, strains assigned to the same cluster seemed to have similar resistance patterns, suggesting that further genotyping of the *S. aureus* strains may assist in designing more effective clinical treatment regimens.

Certain limitations were noted within this study. Firstly, more detailed clinical information was difficult to obtain, especially for outpatients, which account for the majority of patients; therefore, the study was limited in its ability to analyze the effect of additional risk factors for *S. aureus* carriage or MRSA colonization, including premature birth, duration of hospital stay, mother carriage status, history of antibiotic intake, etc. Secondly, inpatient status is strongly correlated with the carriage of MRSA; however, due to the diversity of the hospital environment, we were unable to differentiate MRSA strains based on the various areas in which the inpatients were admitted to; the source of MRSA is worth exploring further. Lastly, we only applied a single method for *S. aureus* typing, which limited access to more specific and detailed prevalent molecular characterization of *S. aureus*.

In summary, the *S. aureus* carriage rate in pediatric feces in Southern China was as high as 20%. MSSA and MRSA exhibited significant differences in genotyping and antimicrobial susceptibility, with ST59 and ST45 emerging as two major MRSA clones and ST188 as the most prevalent MSSA clone. Antibiotic resistance patterns of *S. aureus* were also found to be closely associated with specific STs. These findings clarify the colonization dynamics and molecular epidemiology of *S. aureus* from child feces in Southern China and suggest an urgent need to strengthen the surveillance programs in this region, while also providing important information regarding the prevention and treatment of *S. aureus* infection.

## Data Availability Statement

The datasets generated for this study can be found in the https://pubmlst.org/bigsdb?db=pubmlst_saureus_seqdef&page=downloadProfiles&scheme_id=1.

## Ethics Statement

The studies involving human participants were reviewed and approved by the research ethics committee of the Guangzhou women and children's medical center (registration no. 2016081029). Written informed consent to participate in this study was provided by the participants' legal guardian/next of kin.

## Author Contributions

ZZ and YL initiated and designed the study. XA, FG, SY, BL, JM, ZX, XC, and ZL performed the experiments and/or analyzed the data. XA wrote the draft. HY, ZO, SG, ZZ, and YL revised the manuscript. All authors have approved the final version.

## Conflict of Interest

The authors declare that the research was conducted in the absence of any commercial or financial relationships that could be construed as a potential conflict of interest.

## Supplementary Material

Figure S1 Antibiotic resistance of *S. aureus* isolates from pediatric feces linked to sequence types, as illustrated by the tri-color scale.
