# Supplementary material for: Prevalence, Characterization, and Drug Resistance of Staphylococcus Aureus in Feces From Pediatric Patients in Guangzhou, China
Source: Front Med (Lausanne). 2020 Apr 24;7:127. doi: 10.3389/fmed.2020.00127 (PMC7193981; doi:10.3389/fmed.2020.00127)
Supplement: Supplementary file 1 [file Table_1.docx]

**Table S1** **|** Novel sequence types of *S. aureus* isolated from pediatric patients.

| STs | CCs | Types | Strains | arcC | aroE | glpF | gmk | pta | tpi | yqiL |
| --- | --- | --- | --- | --- | --- | --- | --- | --- | --- | --- |
| ST5307 | CC188 | MSSA | 1 | 3 | 1 | 1 | 8 | 646 | 1 | 1 |
| ST5308 | CC7 | MSSA | 1 | 5 | 788***** | 1 | 4 | 4 | 6 | 3 |
| ST5309 | CC7 | MSSA | 1 | 637***** | 4 | 1 | 4 | 4 | 6 | 3 |
| ST5310 | CC45 | MSSA | 1 | 10 | 14 | 721***** | 6 | 10 | 3 | 2 |
| ST5311 | CC45 | MSSA | 1 | 10 | 14 | 8 | 6 | 10 | 588***** | 2 |
| ST5312 | CC15 | MSSA | 1 | 13 | 13 | 1 | 1 | 654***** | 11 | 13 |
| ST5313 | CC1 | MSSA | 1 | 1 | 506 | 1 | 1 | 1 | 1 | 1 |
| ST5314 | CC2483 | MSSA | 1 | 151 | 789***** | 215 | 34 | 175 | 180 | 169 |
| ST5315 | CC7 | MSSA | 1 | 5 | 431 | 1 | 4 | 4 | 6 | 3 |
| ST5316 | CC630 | MSSA | 1 | 3 | 3 | 1 | 1 | 4 | 4 | 709***** |
| ST5317 | CC59 | MRSA | 1 | 19 | 23 | 15 | 2 | 655***** | 20 | 15 |
| ST5318 | CC188 | MSSA | 1 | 3 | 1 | 61 | 8 | 1 | 1 | 1 |
| ST5319 | CC101 | MSSA | 1 | 638***** | 1 | 14 | 15 | 11 | 19 | 3 |
| ST5320 | CC59 | MRSA | 1 | 19 | 23 | 15 | 2 | 19 | 20 | 710***** |
| ST5321 | CC5 | MSSA | 1 | 639***** | 4 | 1 | 4 | 12 | 121 | 10 |
| ST5322 | CC7 | MSSA | 1 | 5 | 4 | 1 | 403 | 4 | 6 | 3 |
| ST5323 | CC398 | MSSA | 1 | 3 | 790***** | 19 | 2 | 20 | 26 | 39 |
| ST5324 | CC5 | MSSA | 1 | 1 | 4 | 1 | 413***** | 12 | 121 | 10 |
| ST5325 | CC188 | MSSA | 1 | 3 | 1 | 1 | 414***** | 1 | 1 | 1 |
| ST5326 | CC188 | MSSA | 1 | 3 | 791***** | 1 | 8 | 1 | 1 | 1 |
| ST5327 | CC5 | MSSA | 1 | 1 | 4 | 1 | 4 | 359 | 1 | 10 |
| ST5328 | CC88 | MSSA | 1 | 22 | 1 | 14 | 23 | 12 | 589***** | 31 |
| ST5329 | CC1 | MSSA | 1 | 1 | 1 | 1 | 1 | 1 | 1 | 711***** |
| ST5330 | CC121 | MSSA | 1 | 6 | 5 | 16 | 415***** | 7 | 14 | 34 |
| ST5353 | CC188 | MSSA | 2 | 3 | 131 | 1 | 8 | 1 | 1 | 1 |

*****: Newly assigned alleles on seven housekeeping genes of *S. aureus*.
