# Supplementary figures and images for: Prevalence, Characterization, and Drug Resistance of Staphylococcus Aureus in Feces From Pediatric Patients in Guangzhou, China
Source: Front Med (Lausanne). 2020 Apr 24;7:127. doi: 10.3389/fmed.2020.00127 (PMC7193981; doi:10.3389/fmed.2020.00127)

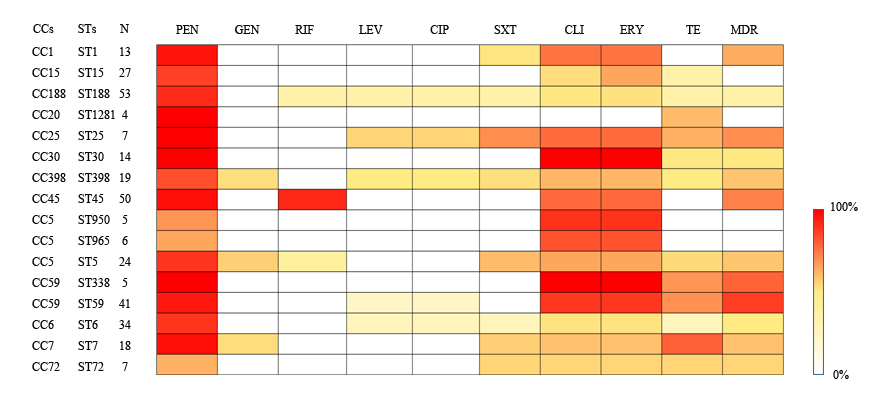

Supplement: Figure S1 — Antibiotic resistance of S. aureus isolates from pediatric feces linked to sequence types, as illustrated by the tri-color scale. [file Image_1.TIF]
